# Health Utility Weighting of the Modified Rankin Scale: A Systematic Review and Meta-analysis

**DOI:** 10.1001/jamanetworkopen.2020.3767

**Published:** 2020-04-29

**Authors:** Alexander D. Rebchuk, Zoe R. O’Neill, Elena K. Szefer, Michael D. Hill, Thalia S. Field

**Affiliations:** 1 Faculty of Medicine, The University of British Columbia, Vancouver, British Columbia, Canada; 2 Faculty of Medicine, McGill University, Montreal, Quebec, Canada; 3 Emmes Canada, Vancouver, British Columbia, Canada; 4 Hotchkiss Brain Institute, Cumming School of Medicine, University of Calgary, Calgary, Alberta, Canada; 5 Department of Clinical Neurosciences, Cumming School of Medicine, University of Calgary, Calgary, Alberta, Canada; 6 Djavad Mowafaghian Centre for Brain Health, The University of British Columbia, Vancouver, British Columbia, Canada; 7 Vancouver Stroke Program, The University of British Columbia, Vancouver, British Columbia, Canada

## Abstract

**Importance:**

The utility-weighted modified Rankin Scale (UW-mRS) has been proposed as a patient-centered alternative primary outcome for stroke clinical trials. However, to date, there is no clear consensus on an approach to weighting the mRS.

**Objective:**

To characterize the between-study variability in utility weighting of the mRS in a population of patients who experienced stroke and its implications when applied to the results of a clinical trial.

**Data Sources:**

In this systematic review and meta-analysis, MEDLINE, Embase, and PsycINFO were searched from January 1987 through May 2019 using major search terms for stroke, health utility, and mRS.

**Study Selection:**

Original research articles published in English were reviewed. Included were studies with participants 18 years or older with ischemic or hemorrhagic stroke, transient ischemic attack, or subarachnoid hemorrhage, with mRS scores and utility weights evaluated concurrently. A total of 5725 unique articles were identified. Of these, 283 met criteria for full-text review, and 24 were included in the meta-analysis.

**Data Extraction and Synthesis:**

PRISMA guidelines for systematic review were followed. Data extraction was performed independently by multiple researchers. Data were pooled using mixed models.

**Main Outcomes and Measures:**

The mean utility weights and 95% CIs were calculated for each mRS score and health utility scale. Geographic differences in weighting for the EuroQoL 5-dimension (EQ-5D) and Stroke Impact Scale–based UW-mRS were explored using inverse variance–weighted linear models. The results of 18 major acute stroke trials cited in current guidelines were then reanalyzed using the UW-mRS weighting scales identified in the systematic review.

**Results:**

The meta-analysis included 22 389 individuals; the mean (SD) age of participants was 65.9 (4.0) years, and the mean (SD) proportion of male participants was 58.2% (7.5%). For all health utility scales evaluated, statistically significant differences were observed between the mean utility weights by mRS score. For studies using an EQ-5D–weighted mRS, between-study variance was higher for worse (mRS 2-5) compared with better (mRS 0-1) scores. Of the 18 major acute stroke trials with reanalyzed results, 3 had an unstable outcome when using different UW-mRSs.

**Conclusions and Relevance:**

Multiple factors, including cohort-specific characteristics and health utility scale selection, can influence mRS utility weighting. If the UW-mRS is selected as a primary outcome, the approach to weighting may alter the results of a clinical trial. Researchers using the UW-mRS should prospectively and concurrently obtain mRS scores and utility weights to characterize study-specific outcomes.

## Key Points

**Question:**

Is a preexisting health utility–weighted outcome scale suitable for use in a clinical trial, or is a study-specific approach more appropriate?

**Findings:**

Among 24 studies including 22 389 individuals, this systematic review and meta-analysis found statistically significant between-study differences for studies reporting utility weighting of the modified Rankin Scale. When applied to the results of major acute stroke trials, different study-specific utility weights led to instability of the primary outcome in some cases.

**Meaning:**

Utility weighting and its interpretation vary based on both the scale used for weighting and the study cohort; furthermore, the choice of utility-weighted outcome scale may alter a trial’s outcome.

## Teaser

This systematic review and meta-analysis characterizes the between-study variability in health utility weighting of the modified Rankin Scale in a population of patients with stroke and its implications when applied to the results of a clinical trial.

## Introduction

The modified Rankin Scale (mRS) is an efficient, reliable, and simple functional outcome measure that is widely used as a primary end point for clinical trials in acute stroke.^1,2,3^ However, as an ordered categorical scale, it may not reflect potentially unequal differences in perceived quality of life associated with certain 1-point shifts vs others. For example, the ordering of outcomes as scores rated from 0 (no residual symptoms) to 6 (death) does not reflect the fact that some individuals may prefer death (mRS score 6) to being bedridden, incontinent, and completely dependent on others (mRS score 5). To accommodate for an improved focus on patient-centered outcomes in clinical trials, the Stroke Treatment Academic Industry Roundtable (STAIR VII) recommended the development of a utility-weighted mRS (UW-mRS) that weights the mRS against a health utility scale.^4,5^ The UW-mRS is increasingly used as an end point in clinical stroke trials. Notably, it was a co–primary end point in the DAWN trial,^6^ and it is the primary outcome in multiple actively enrolling randomized clinical stroke trials.^7,8,9,10^

Health utility, defined as the desirability of a specific health outcome, allows for comparisons of health-related quality of life across an array of clinical settings.^11^ Health utility weights, hereafter referred to as utility weights, reflect the spectrum between perfect health (a score of 1) and outcomes worse than death (where death is a score of 0 and negative values indicate an outcome worse than death). Potential challenges in adopting a one-size-fits-all approach to utility weighting for the mRS include differences in elicitation methods (time trade-off or person trade-off techniques),^11^ selection of an appropriate health utility scale for weighting,^12^ variations in region-specific norms,^13,14^ and between-person differences in preference weighting for a given mRS score.^15,16^ For example, the incorrect application of region-specific norms can substantially alter utility weighting and in turn may influence economic assessments.^17^

The literature was systematically reviewed for all studies that concurrently reported mRS scores and utility weights in stroke survivors, with the aim of exploring potential implications in using and interpreting a UW-mRS in the poststroke population. First, differences in utility weighting were examined between studies that used different health utility scales. Next, interstudy variance in utility weighting was compared between studies using the same health utility scale, and the associations of geographically specific tariffs were explored. In addition, EuroQoL 5-dimension (EQ-5D)–weighted UW-mRSs identified by the systematic review were retrospectively applied to major superiority design acute clinical stroke trials to assess how the outcome of each trial might be interpreted.

## Methods

### Search Strategy

In this systematic review and meta-analysis, MEDLINE, Embase, and PsycINFO were searched from January 1987 through May 2019 using major search terms for stroke, health utility, and modified Rankin Scale. The literature search strategy was based on previous systematic reviews and meta-analyses.^18,19,20^ The reference lists of included articles were manually searched for additional studies. The complete search strategy and a full list of search terms are included in the eMethods in the Supplement.

The Preferred Reporting Items for Systematic Reviews and Meta-Analyses (PRISMA) guidelines for systematic review were followed.^21^ The protocol was registered with the International Prospective Register of Systematic Reviews (PROSPERO).^22^

### Eligibility Criteria

Study eligibility criteria were as follows: (1) participants had an ischemic stroke, hemorrhagic stroke, transient ischemic attack, or subarachnoid hemorrhage; (2) participants were 18 years or older; (3) mRS scores and utility weights were evaluated concurrently; (4) utility weights were mapped to mRS scores; and (5) the scale used to measure health utility was reported. Only original research articles published in English were reviewed. In case of missing data or matters of clarification, the corresponding author was contacted for additional details.

### Data Extraction and Risk of Bias

Two of us (A.D.R. and Z.R.O.) independently screened titles and abstracts of all articles obtained in the initial database search. Those that met preliminary inclusion criteria were then screened by full-text review against the eligibility criteria by the same 2 authors. Any disagreement was resolved by consensus.

Data were extracted from eligible articles using a data collection template (eMethods in the Supplement). Extracted were article and study characteristics, participant demographics, clinical characteristics, health utility scale, mRS scores, and utility weights. Studies were evaluated with a risk of bias tool adapted from work by Gupta et al,^23,24^ which considered selection, detection, reporting, risk of attrition, and confounding biases (eTable 4 in the Supplement).

### Statistical Analysis

Utility weight was defined as the mean health utility weight reported for a given mRS score. Because previous literature suggested that mRS utility weights remained stable over time,^25^ we combined UW-mRSs obtained at different times after stroke. All utility weights were converted to a scale of 0 (death) to 1 (perfect health) to simplify interscale comparisons, with a utility weight of 0 assigned to mRS 6 (death).^11^ Because most studies did not differentiate mRS scores and health utility outcome data by stroke type, we combined all stroke types.

Data were pooled using mixed models. The EQ-5D 3-Level (EQ-5D-3L) and EQ-5D 5-Level (EQ-5D-5L) models were treated as a single scale (EQ-5D) because we confirmed that there were no statistically significant differences between the mean EQ-5D-3L and EQ-5D-5L health utility weights for each mRS score.^26^ For the 36-Item Short Form Survey (SF-36), the social function (SF-36-SF) and physical function (SF-36-PF) subcomponents were separated. The mean utility weights and 95% CIs were calculated for each mRS score and health utility scale.

For the EQ-5D, the only health utility scale for which multiple studies reported the mean and SE of the mean for each mRS score, an inverse variance–weighted linear model was fit with the mean utility weight as the outcome, mRS score as a categorical predictor, and study as a random intercept term. Inverse variance weighting was used to account for differences in variances of each study so that studies with smaller variances for utility weights were more highly weighted in the analysis.

To assess differences between the mean utility weight by mRS score, an *F* test was conducted. Tukey tests for pairwise differences in the mean utility weights between mRS scores were conducted. To assess whether there were differences in the EQ-5D by geography, continent was included as a fixed effect in the model, and a type III *F* test for differences in the mean utility weight by continent was conducted.

Data were sufficient to evaluate differences in variance between EQ-5D–weighted mRS scores with the Levene test. Then, the Levene test was repeated examining differences in variance using different dichotomized mRS cut points (0-1 vs 2-5, 0-2 vs 3-5, and 0-3 vs 4-5).

Only 1 study had the necessary data available for hypothesis testing for each of the following instruments: SF-36-PF,^27^ SF-36-SF,^27^ World Health Organization Global Burden of Disease Project,^28^ Patient-Reported Outcomes Measurement Information System–Physical Function,^29^ Quality of Life in Neurological Disorders,^29^ Health-Related Quality of Life in Stroke Patients,^30^ and Assessment of Quality of Life.^15^ For these health utility scales, an *F* test was conducted to compare the mean utility weights at each mRS score, and Tukey tests for pairwise differences were conducted to compare pairwise differences at all mRS scores.

To assess differences in the 1 study^31^ that reported Stroke Impact Scale (SIS)-16 scores, *F* tests and Tukey pairwise comparisons were conducted. To model SIS domain values by mRS score, an inverse variance–weighted linear model was fit with the mean domain value as the outcome; mRS score, SIS domain, and the interaction between mRS score and SIS domain as categorical predictors; and study as a random intercept term for all domains other than SIS-16. To test for differences in mRS score by domain, *F* tests were conducted. For SIS domains other than SIS-16, continent and the interaction between continent and domain were included as categorical fixed effects in the model. To test for differences in the mean domain values by continent, *F* tests were conducted.

In addition, different EQ-5D–weighted UW-mRSs identified in the systematic review were applied to the results of major acute stroke trials. This method of reanalyzing clinical trial data using the UW-mRS has been published previously.^6^ Clinical trials were selected if they reported group results from all 7 mRS scores, used the mRS as their primary outcome, and were considered in Canadian Best Practices^32^ or American Heart Association/American Stroke Association^33,34^ guidelines for acute ischemic stroke. We identified 18 eligible major acute stroke trials and converted their primary outcome mRS scores to the EQ-5D–weighted UW-mRS scores identified by the systematic review.

All data analyses were conducted in SAS (version 9.4; SAS Institute Inc) and MATLAB (version R2019a; MathWorks). Pairwise *F* tests and Tukey tests were conducted by hand with formulas. Statistical significance was set at 2-sided *P* < .05.

## Results

**Figure 1.**
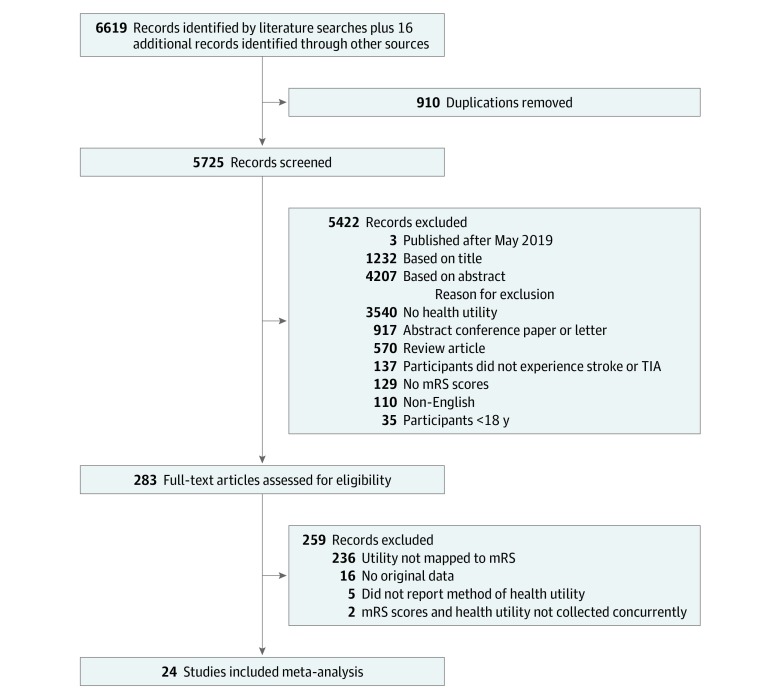
Preferred Reporting Items for Systematic Reviews and Meta-Analyses (PRISMA) Diagram mRS indicates modified Rankin Scale; TIA, transient ischemic attack.

The literature search was last repeated on May 10, 2019. The search strategy initially identified 6619 articles; 910 were duplicates. An additional 16 articles were identified through screening the reference lists. In total, 5725 unique articles underwent formal screening. Based on titles and abstracts, 283 articles met criteria for full-text review. Articles were most frequently excluded during screening (3540 [61.8%]) for failing to mention health utility. Of articles undergoing full-text review, 24 met inclusion criteria and were included in the meta-analysis (Figure 1).

The meta-analysis included 22 389 patients from 41 countries across 6 continents (North America, South America, Europe, Asia, Africa, and Australia). The mean (SD) age of participants was 65.9 (4.0) years, and the mean (SD) proportion of male participants was 58.2% (7.5%). The median (interquartile range [IQR]) time from stroke onset to outcome determination was 90 (82-180) days. Reported stroke types included combined ischemic and hemorrhagic (14 studies^13,15,25,27,28,30,35,36,37,38,39,40,41,42^), ischemic (6 studies^14,16,31,43,44,45^), and hemorrhagic (3 studies^43,46,47^). Two studies^25,44^ included individuals with transient ischemic attack. The median (IQR) sample size was 400 (180-847). The median (IQR) proxy completion rate (eg, by caregiver) was 22.8% (10.6%-34.0%), but 13 studies^25,27,28,31,35,38,39,41,42,43,46,47^ did not report proxy completion rates. Demographic and baseline clinical data are summarized in eTable 1 in the Supplement.

Only studies that reported the sample size in addition to the mean and SD or SE of the mean for each mRS score and utility weights at each mRS score were included in the meta-analysis. Nine studies^13,16,25,36,37,43,44,45,47^ using the EQ-5D (n = 9607), 5 studies^31,38,39,40,48^ using the SIS (n = 777), and 1 study each using the SF-36-PF (n = 278),^27^ SF-36-SF (n = 278),^27^ World Health Organization Global Burden of Disease Project (n = 54),^28^ Patient-Reported Outcomes Measurement Information System–Physical Function (n = 236),^29^ Quality of Life in Neurological Disorders (n = 236),^29^ Health-Related Quality of Life in Stroke Patients (n = 103)^30^ and Assessment of Quality of Life (n = 1523)^15^ met these criteria.

**Figure 2.**
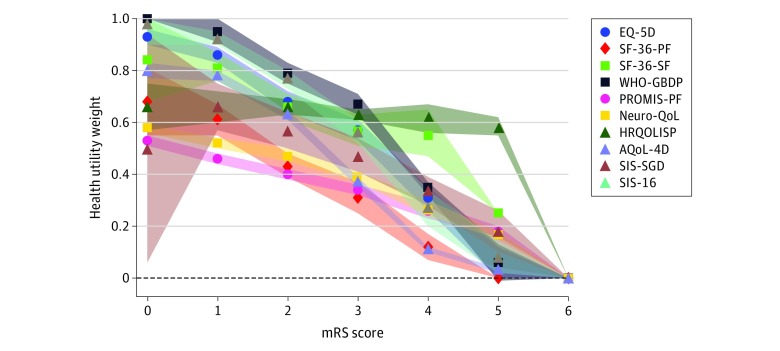
Mean Health Utility Weights by Modified Rankin Scale (mRS) Score for Each Utility Scale, With 95% CIs AQoL-4D indicates Assessment of Quality of Life; EQ-5D, EuroQoL 5-dimension; HRQOLISP, Health-Related Quality of Life in Stroke Patients; Neuro-QoL, Quality of Life in Neurological Disorders; PROMIS-PF, Patient-Reported Outcomes Measurement Information System–Physical Function; SF-36-PF, 36-Item Short Form Survey (SF-36) physical function; SF-36-SF, SF-36 social function; SIS-16, Stroke Impact Scale 16; SIS-SGD, SIS–Stroke Global Disability; and WHO-GBDP, World Health Organization Global Burden of Disease Project. The dashed line represents quality of life equivalent to death; below the dashed line represents quality of life worse than death; shaded areas represent the 95% CIs. Data are presented in eTables 2 and 3 in the Supplement.

Statistically significant differences were observed between the mean utility weights by mRS score for all health utility scales evaluated (Figure 2). For studies using an EQ-5D–weighted mRS score, between-study variance was higher for worse (mRS 2-5) compared with better (mRS 0-1) scores. Of the 18 major acute stroke trials with reanalyzed results, 3 trials^49,50,51^ had an unstable outcome when using different UW-mRSs. With the EQ-5D, there were pairwise differences between all mRS scores. Other health utility scales were variable in distinguishing pairwise differences in utility weights between mRS scores (eTable 2 in the Supplement). For SIS domains, a statistically significant difference was found in the mean domain score by mRS score for every domain except communication (eTable 3 in the Supplement).

For EQ-5D–generated utility weights, no differences were found in utility weighting between continents. However, this analysis was limited to geographic information from Europe (5 studies), Asia (2 studies), and undifferentiated regions (2 studies). A difference in SIS-generated utility weights by continent was observed for the emotion, social participation, and stroke global disability domains. Estimated SIS utility weights were generally higher for South America compared with Europe.

**Figure 3.**
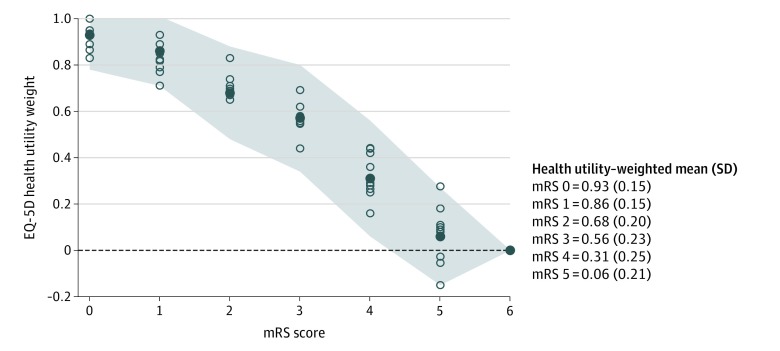
Modified Rankin Scale (mRS) Utility Weights Derived From the EuroQoL 5-Dimension (EQ-5D) Individual studies (n = 9) are represented by blue circles. The grand means (solid blue circles) and SDs (shaded area) of all included studies are shown. The dashed line represents quality of life equivalent to death; below the dashed line represents quality of life worse than death.

Based on the Levene test, heterogeneity of variance (*P* = .06) between each mRS score for EQ-5D–weighted mRS scores was not statistically significant (Figure 3). In the dichotomized analysis, there was a statistically significant difference in variance between mRS scores of 0-1 vs 2-5 and 0-2 vs 3-5, with no statistically significance difference for 0-3 vs 4-5.

When EQ-5D–weighted UW-mRSs were used to reanalyze 18 major acute stroke trials, 15 trials^6,52,53,54,55,56,57,58,59,60,61,62,63,64,65^ had a stable result (ie, positive [*P* < .05] or neutral [*P* > .05] result for the primary outcome remained the same) (Table). Three trials, INTERACT2,^51^ REVASCAT,^50^ and THRACE,^49^ had a variable result dependent on which UW-mRS was used to replace the primary outcome. Four UW-mRSs^16,37,43,47^ had differences in the primary outcome for more than 1 trial. These 4 scales had higher utility weights compared with our calculated mean utility weights for mRS scores 4 and 5.

**Table.** Reanalysis of Primary Outcomes From 18 Major Acute Stroke Trials^a^

Trial	Analysis method	Primary outcome^b^	UW-mRSs with congruent results, %	UW-mRSs with incongruent results^c^
ATACH-2^52^	Dichotomized	Neutral	100	NA
DAWN^6^	Dichotomized	Positive^d^	100	NA
DEFUSE-3^53^	Shift	Positive^d^	100	NA
ECASS II^54^	Dichotomized	Neutral	100	NA
ECASS III^55^	Dichotomized	Positive^e^	0	Rivero-Arias et al,^25^ 2010; Dijkland et al,^16^ 2018; Rangaraju et al,^43^ 2017; Golicki et al,^36^ 2015; Whynes et al,^13^ 2013; Hattori et al,^37^ 2012; Wang et al,^44^ 2014; Dewilde et al,^45^ 2019; Sallinen et al,^47^ 2019
ENCHANTED^56^	Dichotomized	Neutral	100	NA
ESCAPE^57^	Shift	Positive^d^	100	NA
FAST-MAG^58^	Shift	Neutral	100	NA
IMS III^59^	Dichotomized	Neutral	100	NA
INTERACT2^51^	Dichotomized	Neutral^f^	44.4	Rivero-Arias et al,^25^ 2010; Golicki et al,^36^ 2015; Whynes et al,^13^ 2013; Wang et al,^44^ 2014; Dewilde et al,^45^ 2019
ISAT^60^	Dichotomized	Positive^g^	100	NA
MELT^61^	Dichotomized	Neutral	100	NA
MR CLEAN^62^	Shift	Positive^e^	100	NA
NOR-TEST^63^	Dichotomized	Neutral	100	NA
PROACT2^64^	Dichotomized	Positive^e^	0	Rivero-Arias et al,^25^ 2010; Dijkland et al,^16^ 2018; Rangaraju et al,^43^ 2017; Golicki et al,^36^ 2015; Whynes et al,^13^ 2013; Hattori et al,^37^ 2012; Wang et al,^44^ 2014; Dewilde et al,^45^ 2019; Sallinen et al,^47^ 2019
REVASCAT^50^	Shift	Positive^e^	55.5	Dijkland et al,^16^ 2018; Rangaraju et al,^43^ 2017; Hattori et al,^37^ 2012; Sallinen et al,^47^ 2019
SWIFT PRIME^65^	Shift	Positive^d^	100	NA
THRACE^49^	Dichotomized	Positive^e^	11.1	Rivero-Arias et al,^25^ 2010; Dijkland et al,^16^ 2018; Rangaraju et al,^43^ 2017; Golicki et al,^36^ 2015; Whynes et al,^13^ 2013; Hattori et al,^37^ 2012; Dewilde et al,^45^ 2019; Sallinen et al,^47^ 2019

Abbreviations: NA, not applicable; UW-mRSs, utility-weighted modified Rankin Scales.

^a^ Raw mRS scores were converted to the EuroQoL 5-dimension–based UW-mRS using ordinal analysis. All dichotomized analyses used mRS cutoff scores of 0-2 vs 3-6.

^b^ Positive indicates *P* < .05. Neutral indicates non–statistically significant (*P* > .05).

^c^ The sources in this column indicate which UW-mRSs, derived from the EQ-5D, identified in our systematic review had results that were incongruent with the reported results of the trial.

^d^ *P* < .001.

^e^ *P* < .05.

^f^ *P* < .10.

^g^ *P* < .01.

## Discussion

The UW-mRS is an increasingly popular primary outcome in randomized clinical trials for acute stroke as a means to incorporate patient preferences. However, despite its emergent role, consensus on the approach to utility weighting is lacking. This work highlights important considerations in using a UW-mRS to reflect a patient-centered approach. First, utility weighting varies based on the cohort and the choice of health utility scale. Second, these differences in weighting may potentially alter the outcome of a clinical trial. Substantial differences were found in utility weighting of the mRS, both when different scales were used and between studies where the same scale was used. As expected, in major acute stroke trials with marginally positive or neutral results,^49,66,67^ use of different study-specific weighting regimens resulted in instability around the primary outcome.

Most of the 24 articles identified in the systematic review used the EQ-5D to generate utility weights. Between-study differences were found in utility weighting using the EQ-5D, particularly with worse functional outcomes. Sociocultural and demographic factors, medical comorbidities, and personal values may have a greater influence on perceived quality of life (and perception of death as a more acceptable state than total dependence on others) in more severely disabled patients, which may in part explain this increased variance.^14,17,68,69^ These differences may contribute to within-cohort heterogeneity in addition to between-study differences. A subanalysis of the MR CLEAN thrombectomy trial showed substantial interindividual variability for EQ-5D weighting of mRS scores and reduced statistical efficiency compared with an ordinal mRS outcome.^16^ An important consequence of this variability is that different UW-mRSs may alter the outcome of clinical trials. In our analysis, we observed that UW-mRSs with higher utility weighting for severely disabled outcomes when applied to 18 major acute stroke trials were likely to lead to a neutral (ie, non–statistically significant) trial result.

Although the mRS score is a universally accepted outcome for major acute stroke trials, use of a concurrent health utility scale may more fully capture changes important to survivors, whereas the mRS cannot. In addition to its implicit value statement in ranking death as the worst possible outcome, the mRS may also be insufficiently sensitive to important functional differences altering quality of life. For example, the EQ-5D may be more responsive than the mRS to stroke survivors’ perceptions of functional changes.^67^ However, these minimally important differences may vary by cohort^49,70,71^; when choosing a health utility scale, it is important that it reflects the needs and values of a particular study population. For example, in an exclusively minor stroke and transient ischemic attack cohort, where issues with fatigue, cognition, and mood may be most important for quality of life,^68^ the EQ-5D (which does not capture cognition) may not be an optimal choice for health utility weighting.

The variability of health utility weighting observed in this study provides evidence to support prior recommendations that clinical trials using a UW-mRS should prospectively and concurrently obtain both the mRS scores and health utility weights to establish trial-specific weighted scales.^14,25^ However, our findings also raise the broader question of whether a weighted score is the best approach to incorporating patient preferences into trial results; it may be more informative to simply provide the mRS score and a health utility weight separately as co-primary outcomes. Using both outcomes separately allows investigators to report functional differences using the clinically interpretable and reliable mRS alongside a contextually appropriate health utility scale to characterize meaningful differences in patient quality of life.

### Limitations

This study has limitations. First, the median proxy completion rate was 22.8%, which may have altered the results of our study. Although proxies, such as family members, help to provide health utility from aphasic or severely disabled patients, they may rate the patient’s health utility more negatively than would the patients themselves.^72,73^ Our high proxy completion rate may have decreased mRS scores, especially for severely disabled patients. Second, combining the EQ-5D-3L and EQ-5D-5L is a potential limitation of this analysis because the EQ-5D-5L generates lower mean utility weights than the EQ-5D-3L, and the EQ-5D-5L has smaller score ranges.^74,75^ Although this limitation could have potentially caused our combined EQ-5D to overestimate utility weights, we found for our data set that the EQ-5D-3L and EQ-5D-5L utility weights at each mRS score did not differ statistically significantly. Third, in keeping with prior research,^25^ we assumed that poststroke utility weights remained stable over time. However, a recent subanalysis of the AVERT rehabilitation trial demonstrated statistically significant within-patient variability in utility weights between 3 and 12 months after stroke in those whose mRS score remained stable over that period.^15^ It is possible and even likely that survivors may become more accepting of their new normal over time or may be experiencing incremental gains not measurable using the mRS.^70,76^ In addition, we were unable to examine time-specific differences in utility weighting in this study given the limitations of the data, but we believe that this aspect is also worthy of further prospective study.

## Conclusions

Utility weighting of the mRS depends on multiple factors, including cohort-specific characteristics and the health utility scale used. The choice of weighting may alter the results of a clinical trial. From this study’s findings, it appears that researchers using the UW-mRS should derive a trial-specific score or should consider simply reporting both the mRS score and utility weights as separate co–primary outcomes.

## Notes

Supplement. **eMethods.** Supplemental Methods **eTable 1.** Aggregate Demographic Data of Included Studies **eTable 2.** Mean Utility Weights by mRS Level (UW-mRS), With Associated 95% CIs and Number of Included Patients for Each Health Utility Scale **eTable 3.** Mean Stroke Impact Scale (SIS) Utility Weights by mRS Level, With Associated 95% CIs, Number of Patients, and Number of Studies Contributing Data, for Each SIS Domain **eTable 4.** Risk of Bias Results for Included Articles
