# Supplementary material for: Health Utility Weighting of the Modified Rankin Scale: A Systematic Review and Meta-analysis
Source: JAMA Netw Open. 2020 Apr 29;3(4):e203767. doi: 10.1001/jamanetworkopen.2020.3767 (PMC7191324; doi:10.1001/jamanetworkopen.2020.3767)
Supplement: Supplement. — eMethods. Supplemental Methods eTable 1. Aggregate Demographic Data of Included Studies eTable 2. Mean Utility Weights by mRS Level (UW-mRS), With Associated 95% CIs and Number of Included Patients for Each Health Utility Scale eTable 3. Mean Stroke Impact Scale (SIS) Utility Weights by mRS Level, With Associated 95% CIs, Number of Patients, and Number of Studies Contributing Data, for Each SIS Domain eTable 4. Risk of Bias Results for Included Articles [file jamanetwopen-3-e203767-s001.pdf]

# Supplementary Online Content

Rebchuk AD, O'Neill ZR, Szefer EK, Hill MD, Field TS. Health utility weighting of the modified Rankin Scale: a systematic review and meta-analysis. *JAMA Netw Open*. 2020;3(4):e203767. doi:10.1001/jamanetworkopen.2020.3767

**eMethods.** Supplemental Methods

**eTable 1.** Aggregate Demographic Data of Included Studies

**eTable 2.** Mean Utility Weights by mRS Level (UW-mRS), With Associated 95% CIs and Number of Included Patients for Each Health Utility Scale

**eTable 3.** Mean Stroke Impact Scale (SIS) Utility Weights by mRS Level, With Associated 95% CIs, Number of Patients, and Number of Studies Contributing Data, for Each SIS Domain

**eTable 4.** Risk of Bias Results for Included Articles

This supplementary material has been provided by the authors to give readers additional information about their work.

© 2020 Rebchuk AD et al. *JAMA Network Open*.

**eMethods.** Supplemental Methods

**Medline Search Strategy**

| | |
|---|---|
| 1 | exp STROKE/ |
| 2 | Cerebrovascular Disorders/ |
| 3 | Carotid Artery Thrombosis/ |
| 4 | Brain Ischemia/ |
| 5 | Cerebral Infarction/ |
| 6 | Ischemic Attack, Transient/ |
| 7 | Vertebral Artery Dissection/ |
| 8 | Cervical Artery Dissection.mp. |
| 9 | Intracranial Artery Dissection.mp. |
| 10 | Cerebral Hemorrhage/ |
| 11 | Subarachnoid Hemorrhage/ |
| 12 | stroke$.mp. |
| 13 | cerebral vasc$.mp. |
| 14 | cerebrovasc$.mp. |
| 15 | cva.mp. |
| 16 | transient isch?emic attack$.mp. |
| 17 | tia$.mp. |
| 18 | or/1-17 |
| 19 | "Quality of Life"/ |
| 20 | Health Status/ |
| 21 | Health Status Indicators/ |
| 22 | quality of health.mp. |
| 23 | QoL.mp. |
| 24 | SS-QoL.mp. |
| 25 | Stroke Impact Scale.ti,ab. |
| 26 | Stroke Adapted Sickness Impact Profile.ti,ab. |
| 27 | Stroke-Specific Quality of Life Measure.ti,ab. |
| 28 | health related quality of life.mp. |
| 29 | HRQoL.mp. |
| 30 | HRQL.mp. |

© 2020 Rebchuk AD et al. *JAMA Network Open*.

| | |
|---|---|
| 31 | health utility.mp. |
| 32 | "Outcome Assessment (Health Care)"/ |
| 33 | quality adjusted life year$.mp. |
| 34 | Quality-Adjusted Life Years/ |
| 35 | QALY$.mp. |
| 36 | utility weight$.mp. |
| 37 | utility?weight$.mp. |
| 38 | disability weight$.mp. |
| 39 | Disability Evaluation/ |
| 40 | DALY$.mp. |
| 41 | EuroQoL.mp. |
| 42 | Euro-QoL.mp. |
| 43 | Neuro?QoL.mp. |
| 44 | EQ-5D-3L.mp. |
| 45 | EQ-5D-5L.mp. |
| 46 | EQ-5D.mp. |
| 47 | SF-36.mp. |
| 48 | Standard Gamble.mp. |
| 49 | SG.mp. |
| 50 | Time trade?off*.mp. |
| 51 | TTO.mp. |
| 52 | Person trade?off*.mp. |
| 53 | PTO.mp. |
| 54 | Visual Analogue Scale.mp. |
| 55 | VAS.mp. |
| 56 | or/19-55 |
| 57 | mRS$.mp. |
| 58 | modified rankin scale.mp. |
| 59 | 57 or 58 |
| 60 | 18 and 56 and 59 |

**Embase Search Strategy**

| | |
|---|---|
| 1 | exp *cerebrovascular accident/ |

© 2020 Rebchuk AD et al. *JAMA Network Open*.

| | |
|---|---|
| 2 | cva.ti,ab. |
| 3 | stroke$.ti,ab. |
| 4 | exp *brain ischemia/ |
| 5 | *brain infarction/ |
| 6 | cerebral infarction.ti,ab. |
| 7 | exp *cerebrovascular disease/ |
| 8 | *transient ischemic attack/ |
| 9 | tia.ti,ab. |
| 10 | transient isch?emic attack$.ti,ab. |
| 11 | *Cerebral Hemorrhage/ |
| 12 | *Subarachnoid hemorrhage/ |
| 13 | Vertebral Artery Dissection.ti,ab. |
| 14 | Cervical Artery Dissection.ti,ab. |
| 15 | Intracranial Artery Dissection.ti,ab. |
| 16 | (cerebro?vasc: disease* or cerebral vasc: disease*).ti,ab. |
| 17 | or/1-14 |
| 18 | exp "quality of life"/ |
| 19 | exp health status/ |
| 20 | exp health status indicator/ |
| 21 | quality of health.mp. |
| 22 | QoL.mp. |
| 23 | health related quality of life.mp. |
| 24 | SS-QoL.mp. |
| 25 | Stroke Adapted Sickness Impact Profile.ti,ab. |
| 26 | Stroke Impact Scale.ti,ab. |
| 27 | Stroke-Specific Quality of Life Measure.ti,ab. |
| 28 | HRQoL.mp. |
| 29 | HRQL.mp. |
| 30 | health utility.mp. |
| 31 | quality adjusted life year/ |
| 32 | Quality?Adjusted Life Year$.mp. |
| 33 | QALY$.mp. |

© 2020 Rebchuk AD et al. *JAMA Network Open*.

| | |
|---|---|
| 34 | utility weight$.mp. |
| 35 | utility?weight$.mp. |
| 36 | outcome assessment/ |
| 37 | disability weight$.mp. |
| 38 | DALY.mp. |
| 39 | disability evaluation.mp. |
| 40 | Euro?QoL.mp. |
| 41 | Neuro?QoL.mp. |
| 42 | EQ-5D-3L.mp. |
| 43 | EQ-5D-5L.mp. |
| 44 | EQ-5D.mp. |
| 45 | SF-36.mp. |
| 46 | Standard Gamble.mp. |
| 47 | SG.mp. |
| 48 | TTO.mp. |
| 49 | Time trade-off.mp. |
| 50 | Time trade?off$.mp. |
| 51 | Person trade-off.mp. |
| 52 | Person trade?off$.mp. |
| 53 | PTO.mp. |
| 54 | Visual Analogue Scale.mp. |
| 55 | VAS.mp. |
| 56 | or/18-55 |
| 57 | rankin scale/ |
| 58 | modified rankin scale.mp. |
| 59 | mRS.mp. |
| 60 | or/57-59 |
| 61 | 17 and 56 and 60 |

**PsycINFO Search Strategy**

| | |
|---|---|
| 1 | DE “Cerebrovascular Accidents” |
| 2 | DE "Cerebrovascular Disorders" |
| 3 | DE "Cerebral Ischemia" |
| 4 | DE "Cerebral Hemorrhage" |

© 2020 Rebchuk AD et al. *JAMA Network Open*.

| | |
|---|---|
| 5 | stroke |
| 6 | cva |
| 7 | DE "Quality of Life" |
| 8 | DE "Health Care Costs" |
| 9 | DE "Health Care Economics" |
| 10 | DE "Occupational Status" |
| 11 | DE "Treatment Outcomes" |
| 12 | DE "Physical Health Assessment" |
| 13 | DE "Rating Scales" |
| 14 | DE "Surveys" |
| 15 | DE "Evaluation" |
| 16 | DE "Utility Theory" |
| 17 | QoL |
| 18 | health status |
| 19 | Health status indicators |
| 20 | quality of health |
| 21 | health related quality of life |
| 22 | HRQoL |
| 23 | HRQL |
| 24 | health utility |
| 25 | quality adjusted life years |
| 26 | QALY |
| 27 | utility weight |
| 28 | disability weight |
| 29 | EuroQoL |
| 30 | euro-QoL |
| 31 | neuro-QoL |
| 32 | EQ-5D |
| 33 | EQ-5D-3L |
| 34 | EQ-5D-5L |
| 35 | SF-36 |
| 36 | time trade off |
| 37 | TTO |

© 2020 Rebchuk AD et al. *JAMA Network Open*.

| | |
|---|---|
| 38 | person trade off |
| 39 | person tradeoff |
| 40 | PTO |
| 41 | visual analogue scale |
| 42 | VAS |
| 43 | modified rankin scale |
| 44 | mRS |
| 45 | rankin scale |
| 46 | 43 OR 44 OR 45 |
| 47 | Vertebral Artery Dissection |
| 48 | Cervical Artery Dissection |
| 49 | Intracranial Artery Dissection |
| 50 | Cerebral Hemorrhage |
| 51 | Subarachnoid Hemorrhage |
| 52 | cerebrovascular accident |
| 53 | cerebrovascular disorders |
| 54 | transient ischemic attack |
| 55 | 1 OR 2 OR 3 OR 4 OR 5 OR 6 OR 47 OR 48 OR 49 OR 50 OR 51 OR 52 OR 53 OR 54 |
| 56 | s-qol |
| 57 | Stroke Impact Scale |
| 58 | Stroke Adapted Sickness Impact Profile |
| 59 | Stroke-Specific Quality of Life Measure |
| 60 | 7 OR 8 OR 9 OR 10 OR 11 OR 12 OR 13 OR 14 OR 15 OR 16 OR 17 OR 18 OR 19 OR 20 OR 21 OR 22 OR 23 OR 24 OR 25 OR 26 OR 27 OR 28 OR 29 OR 30 OR 31 OR 32 OR 33 OR 34 OR 35 OR 36 OR 37 OR 38 OR 39 OR 40 OR 41 OR 42 or 56 or 57 or 58 or 59 |
| 61 | 46 AND 55 AND 60 |

## Risk of Bias Tool

**Explanation**

Because no standardized tool exists to assess the risk of bias in observational studies (Sanderson et al., 2007) we modified risk of bias tools used in recently published meta-analysis of imaging markers for stroke risk (Gupta et al., 2016; Gupta et al., 2013; Gupta et al. 2015). We generated 10 questions to assess potential selection, detection, reporting, attrition and confounding bias in included studies.

© 2020 Rebchuk AD et al. *JAMA Network Open*.

**Type of bias**

**Selection**

- Were the inclusion and exclusion criteria clearly described?
- Was the sample randomly selected or community-dwelling (to reduce selection bias)?
- Were the rationale for removing outlier data reported?

**Detection**

- Were mRS assessors blinded to the treatment/intervention/experimental status of the patient?

**Reporting**

- Did more than one investigator assess mRS outcomes?
- Structured mRS questionnaire?

**Attrition**

- Were losses to follow-up recorded and reported?

**Confounding**

- Were comormobities reported?
- Was the time post-stroke of data collection reported?
- Were rates of proxy completion given by mRS level?

## Data Collection Form

- Article
  - Title:
  - Author(s):
  - Year:
  - Journal:
  - Link:
- Basic Data
  - Study Type (incl. prospective/retrospective; Randomized vs prospective vs observational):
  - Center (Single center v multi center):
  - Center (Single center v multi center):
  - n:
  - Sex (%female):
  - Mean age (SD):
  - Countries of Pts:
  - Comorbidities (vascular RF, prexisting QoL, prev stroke):
  - Comorbidities (HTN, DM, CKD, Afib, CAD, hyperlipidemia, smoking Hx):
  - Race/Ethnicity:
  - Religion:
  - Employment Status:
  - % proxy completed:
  - QoL scale:
  - Method of Health Utility:
  - TTO or PTO:

© 2020 Rebchuk AD et al. *JAMA Network Open*.

- Who collected HU (e.g. nurse, physician):
- Stroke Data
  - Stroke Type:
  - Time since event:
  - Barthel Index [mean (SD)]:
  - NIHSS:
  - Intervention/Tx:
  - Stroke Def’n (copy):
- Mapping (w/ SDs, CIs)
  - mRS 0:
  - mRS 1:
  - mRS 2:
  - mRS 3:
  - mRS 4:
  - mRS 5:

© 2020 Rebchuk AD et al. *JAMA Network Open*.

**eTable 1.** Aggregate Demographic Data of Included Studies. Mean and standard deviations weighted by sample size of studies reporting data

| Demographic Variable | Mean (SD) | Studies reporting data (n) |
|---|---|---|
| **Previous stroke or TIA** | 21% (4%) | 7 |
| **Co-Morbidities** | | |
| Hypertension | 67% (8%) | 11 |
| Diabetes | 21% (2%) | 7 |
| Atrial fibrillation | 16% (11%) | 8 |
| Hyperlipidemia | 22% (14%) | 5 |
| Coronary artery disease | 20% (4%) | 5 |
| Heart Failure | 5% (4%) | 4 |
| Myocardial infarction | 7% (6%) | 2 |
| Smoking | 43% (7%) | 4 |
| Alcohol use | 31% | 1 |
| **Race/Ethnicity** | | |
| Caucasian | 77% (7%) | 8 |
| Black | 19% (4%) | 6 |
| Asian | 95% (18%) | 2 |
| Mixed | 41.4% | 1 |
| Indigenous | 1.2% | 1 |
| **Employment** | | |
| Retired | 61% (8%) | 3 |
| Full-time | 22% (4%) | 4 |
| Part-time | 8% (3%) | 2 |
| Unemployed | 10% (1%) | 2 |
| Housework | 17% | 1 |

© 2020 Rebchuk AD et al. *JAMA Network Open*.

**eTable 2.** Mean Utility Weights by mRS Level (UW-mRS), With Associated 95% CIs and Number of Included Patients for Each Health Utility Scale. Pairwise testing performed with Tukey's post-hoc, significance levels are denotated by * ($p<0.05$), † ($p<0.01$) and ‡ ($p<0.001$)

| Health Utility Metric | Studies: analyzed / identified | Total Patients (n) | mRS 0 | mRS 1 | mRS 2 | mRS 3 | mRS 4 | mRS 5 | *p* value |
|---|---|---|---|---|---|---|---|---|---|
| **EQ-5D** | 9/12 | 9607 | 0.93 (0.96, 0.90) [n = 3624] | 0.86 (0.89, 0.83) [n = 2376] | 0.68 (0.72, 0.64) [n = 1149] | 0.57 (0.61, 0.53) [n = 957] | 0.31 (0.35, 0.26) [n = 1101] | 0.06 (0.12, 0.00) [n = 400] | <.0001 |
| | | | All pairwise significant†. | | | | | | |
| **SF-36-PF** | 1/2 | 278 | 0.68 (0.81, 0.55) [n = 10] | 0.61 (0.67, 0.55) [n = 61] | 0.43 (0.48, 0.39) [n = 88] | 0.31 (0.37, 0.25) [n = 65] | 0.12 (0.17, 0.07) [n = 52] | 0 (0, 0) [n = 2] | <.0001 |
| | | | All pairwise significant‡, except mRS 0-1, 1-2, 2-3, 3-4 and 4-5. | | | | | | |
| **SF-36-SF** | 1/1 | 278 | 0.84 (1.0, 0.68) [n = 10] | 0.81 (0.87, 0.76) [n = 61] | 0.66 (0.71, 0.61) [n = 88] | 0.57 (0.63, 0.51) [n = 65] | 0.55 (0.64, 0.47) [n = 52] | 0.25 (0.25, 0.25) [n = 2] | <.0001 |
| | | | All pairwise significant‡, except mRS 0-1, 1-2, 2-3, 2-4, and 3-4. | | | | | | |
| **WHO-GBDP** | 1/1 | 54 | 1.0 (1.0, 1.0) [n = 9] | 0.95 (1.0, 0.91) [n = 9] | 0.79 (0.83, 0.75) [n = 9] | 0.67 (0.71, 0.63) [n = 9] | 0.35 (0.37, 0.32) [n = 9] | 0.06 (0.13, -0.01) [n = 9] | <.0001 |
| | | | All pairwise significant*, except mRS 0-1. | | | | | | |
| **PROMIS-PF** | 1/1 | 236 | 0.53 (0.55, 0.51) [n = 50] | 0.46 (0.48, 0.44) [n = 50] | 0.40 (0.42, 0.38) [n = 35] | 0.34 (0.36, 0.32) [n = 41] | 0.26 (0.29, 0.23) [n = 39] | 0.18 (0.20, 0.16) [n = 21] | <.0001 |
| | | | All pairwise significant†. | | | | | | |

© 2020 Rebchuk AD et al. *JAMA Network Open*.

| | | | | | | | | | |
|---|---|---|---|---|---|---|---|---|---|
| **Neuro-QoL** | 1/1 | 236 | 0.58 (0.59, 0.56) [n = 50] | 0.52 (0.54, 0.50) [n = 50] | 0.47 (0.50, 0.45) [n = 35] | 0.39 (0.41, 0.37) [n = 41] | 0.27 (0.30, 0.24) [n = 39] | 0.17 (0.18, 0.15) [n = 21] | <.0001 |
| | | | All pairwise significant‡, except mRS 0-1 and 1-2. | | | | | | |
| **HRQOLISP** | 1/1 | 103 | 0.66 (0.75, 0.57) [n = 4] | - (-, -) [n = 0] | 0.66 (0.69, 0.63) [n = 38] | 0.63 (0.65, 0.61) [n = 25] | 0.62 (0.67, 0.56) [n = 8] | 0.58 (0.62, 0.55) [n = 28] | 0.0078 |
| | | | No significant pairwise differences. | | | | | | |
| **AQoL-4D** | 1/1 | 1523 | 0.80 (0.83, 0.77) [n = 186] | 0.78 (0.80, 0.76) [n = 404] | 0.63 (0.65, 0.61) [n = 415] | 0.37 (0.39, 0.35) [n = 456] | 0.11 (0.12, 0.10) [n = 267] | 0.03 (0.04, 0.02) [n = 195] | <.0001 |
| | | | All pairwise significant‡, except mRS 0-1. | | | | | | |
| **SIS (incl. components)** | 5/5 | 422-777 | See eTable3. | | | | | | |

© 2020 Rebchuk AD et al. *JAMA Network Open*.

**eTable 3.** Mean Stroke Impact Scale (SIS) Utility Weights by mRS Level, With Associated 95% CIs, Number of Patients, and Number of Studies Contributing Data, for Each SIS Domain. Studies legend: Katzan 2017 (þ), Carod-Artal 2008 (‡), Vellone 2013 (*), Carod-Artal 2009 (ƒ) and Duncan 1999 (†).

| SIS Subscale | Total Patients (n) | mRS 0 | mRS 1 | mRS 2 | mRS 3 | mRS 4 | mRS 5 | *p* value |
|---|---|---|---|---|---|---|---|---|
| **Strength** (†§#) | 422 | - (-, -) [n = 0, studies = 0] | 0.76 (0.62, 0.89) [n = 70, studies = 3] | 0.52 (0.40, 0.63) [n = 108, studies = 3] | 0.39 (0.29, 0.49) [n = 102, studies = 3] | 0.31 (0.24, 0.39) [n = 142, studies = 3] | - (-, -) [n = 0, studies = 0] | <.0001 |
| **Hand Function** (†§#) | 422 | - (-, -) [n = 0, studies = 0] | 0.66 (0.47, 0.86) [n = 70, studies = 3] | 0.31 (0.21, 0.42) [n = 108, studies = 3] | 0.11 (0.06, 0.17) [n = 102, studies = 3] | 0.08 (0.05, 0.11) [n = 142, studies = 3] | - (-, -) [n = 0, studies = 0] | <.0001 |
| **Mobility** (†§#) | 422 | - (-, -) [n = 0, studies = 0] | 0.78 (0.67, 0.90) [n = 70, studies = 3] | 0.62 (0.52, 0.72) [n = 108, studies = 3] | 0.49 (0.40, 0.58) [n = 102, studies = 3] | 0.22 (0.16, 0.28) [n = 142, studies = 3] | - (-, -) [n = 0, studies = 0] | <.0001 |
| **ADL/IADL** (†§#) | 422 | - (-, -) [n = 0, studies = 0] | 0.81 (0.71, 0.92) [n = 70, studies = 3] | 0.63 (0.54, 0.73) [n = 108, studies = 3] | 0.45 (0.39, 0.52) [n = 102, studies = 3] | 0.29 (0.22, 0.35) [n = 142, studies = 3] | - (-, -) [n = 0, studies = 0] | <.0001 |
| **Memory/ Thinking** (†‡§#) | 537 | 74.4 (0.32, 1.17) [n = 6, studies = 1] | 0.76 (0.68, 0.83) [n = 111, studies = 4] | 0.77 (0.72, 0.82) [n = 175, studies = 4] | 0.67 (0.60, 0.74) [n = 173, studies = 4] | 0.57 (0.51, 0.62) [n = 303, studies = 4] | 0.38 (0.23, 0.54) [n = 39, studies = 1] | <.0001 |
| **Communication** (†§#) | 422 | - (-, -) [n = 0, studies = 0] | 0.83 (0.72, 0.94) [n = 70, studies = 3] | 0.80 (0.72, 0.88) [n = 108, studies = 3] | 0.68 (0.55, 0.81) [n = 102, studies = 3] | 0.78 (0.70, 0.87) [n = 142, studies = 3] | - (-, -) [n = 0, studies = 0] | 0.3431 |
| **Emotion** (†‡§#) | 777 | 0.76 (0.42, 1.11) [n = 6, studies = 1] | 0.57 (0.51, 0.62) [n = 111, studies = 4] | 0.59 (0.54, 0.64) [n = 175, studies = 4] | 0.51 (0.46, 0.55) [n = 173, studies = 4] | 0.52 (0.48, 0.56) [n = 303, studies = 4] | 0.45 (0.32, 0.58) [n = 39, studies = 1] | 0.0399 |

© 2020 Rebchuk AD et al. *JAMA Network Open*.

| | | | | | | | | |
|---|---|---|---|---|---|---|---|---|
| **Social Participation** (†‡§#) | 777 | 0.59 (0.06, 1.11) [n = 6, studies = 1] | 0.59 (0.52, 66) [n = 111, studies = 4] | 0.53 (0.46, 0.59) [n = 175, studies = 4] | 0.44 (0.38, 50.1) [n = 173, studies = 4] | 0.32 (0.27, 0.36) [n = 303, studies = 4] | 0.15 (0.07, 0.24) [n = 39, studies = 1] | <.0001 |
| **Composite Physical** (†‡§#) | 777 | 0.57 (-0.01, 1.15) [n = 6, studies = 1] | 0.70 (0.62, 0.78) [n = 111, studies = 4] | 0.54 (0.48, 0.60) [n = 175, studies = 4] | 0.40 (0.36, 0.45) [n = 173, studies = 4] | 0.22 (0.18, 0.25) [n = 303, studies = 4] | 0.08 (0.01, 0.15) [n = 39, studies = 1] | <.0001 |
| **Stroke Global Disability** (†‡) | 459 | 0.50 (0.06, 0.94) [n = 6, studies = 1] | 0.66 (0.57, 0.75) [n = 74, studies = 2] | 0.57 (0.50, 0.64) [n = 113, studies = 2] | 0.47 (0.41, 0.54) [n = 113, studies = 2] | 0.34 (0.28, 0.39) [n = 214, studies = 2] | 0.18 (0.10, 0.26) [n = 39, studies = 1] | <.0001 |
| **SIS16** (*) | 672 | 0.98 (0.90, 1.07) [n = 21, studies = 1] | 0.92 (.90, 0.95) [n = 283, studies = 1] | 0.77 (0.73, 0.80) [n = 330, studies = 1] | 0.56 (0.52, 0.61) [n = 218, studies = 1] | 0.27 (0.20, 0.34) [n = 101, studies = 1] | 0.08 (0.02, 0.14) [n = 16, studies = 1] | <.0001 |

© 2020 Rebchuk AD et al. *JAMA Network Open*.

**eTable 4.** Risk of Bias Results for Included Articles

| Title | Journal | Year | *Inclusion and exclusion criteria clearly described?* | *Randomly selected sample or community-dwelling?* | *Rationale for removing outlier data reported?* | *mRS assessors blinded to the status of the patient?* | *Multiple investigator assesses mRS outcomes?* | *Structured mRS questionnaire?* | *Losses to follow-up reported?* | *Comorbidities reported?* | *Time post-stroke reported?* | *Proxy rates by mRS level?* |
|---|---|---|---|---|---|---|---|---|---|---|---|---|
| Utility-Weighted Modified Rankin Scale as Primary Outcome in Stroke Trials: A Simulation Study. | Stroke | 2018 | yes | Randomly selected | no | no | no | no | yes | yes | yes | no |
| Comparison of 3-Month Stroke Disability and Quality of Life across Modified Rankin Scale Categories. | Interventional Neurology | 2017 | yes | Randomly selected | no | yes | no | no | no | no | yes | no |
| Lifetime health effects and medical costs of integrated stroke services - a non-randomized controlled cluster-trial based life table approach. | Cost Effectiveness & Resource Allocation | 2017 | yes | Randomly selected | no | yes | yes | no | yes | yes | yes | no |
| Added Value of Patient-Reported Outcome Measures in Stroke Clinical Practice. | Journal of the American Heart Association | 2017 | no | Community dwelling | no | no | no | yes | yes | no | yes | no |
| Web-Based Assessment of Outcomes After Subarachnoid and Intracerebral Hemorrhage: A New Patient Centered Option for Outcomes Assessment. | Neurocritical Care | 2015 | yes | Community dwelling | no | no | no | yes | yes | yes | yes | no |
| Validity of EQ-5D-5L in stroke. | Quality of Life Research | 2015 | yes | Community dwelling | no | no | no | yes | yes | no | yes | no |
| Testing for differential item functioning within the EQ-5D. | Medical Decision Making | 2013 | yes | Randomly selected | yes | no | no | yes | no | no | yes | yes |
| Medical care for chronic-phase stroke in Japan. | Neurologia Medico-Chirurgica | 2012 | yes | Community dwelling | no | no | no | yes | yes | no | yes | no |
| Mapping the modified Rankin scale (mRS) | Medical Decision | 2010 | yes | Randomly selected | no | no | no | yes | yes | no | yes | no |

© 2020 Rebchuk AD et al. *JAMA Network Open*.

| | | | | | | | | | | | | |
|---|---|---|---|---|---|---|---|---|---|---|---|---|
| measurement into the generic EuroQol (EQ-5D) health outcome. | Making | | | | | | | | | | | |
| Quantifying the value of stroke disability outcomes: WHO global burden of disease project disability weights for each level of the modified Rankin Scale. | Stroke | 2009 | n/a | n/a | no | no | yes | yes | n/a | no | no | n/a |
| The stroke impact scale 3.0: evaluation of acceptability, reliability, and validity of the Brazilian version. | Stroke | 2008 | yes | Randomly selected | no | no | no | no | no | yes | yes (mean varied between participants ) | no |
| Physical and social functioning after stroke: comparison of the Stroke Impact Scale and Short Form-36. | Stroke | 2003 | yes | Community dwelling | no | yes | no | yes | no | no | yes | no |
| Defining post-stroke recovery: implications for design and interpretation of drug trials. | Neuropharmacology | 2000 | yes | Community dwelling | no | no | no | yes | yes | no | yes | no |
| Dependency and health utilities in stroke: Data to inform cost-effectiveness analyses. | European Stroke Journal | 2017 | yes | Randomly selected | no | no | no | no | yes | yes | yes | no |
| Psychometric properties of the German version of the health-related quality of life in stroke patients (HRQOLISP) instrument. | NeuroRehabilitation. | 2013 | yes | Community dwelling | no | no | no | no | no | no | yes | no |
| Psychometric properties of the Italian version of the stroke impact scale 3.0. | European Journal of Cardiovascular Nursing | 2013 | yes | Randomly selected | no | no | no | no | yes | no | yes | no |
| Self-and proxy-report agreement on the stroke impact scale. | Stroke | 2009 | yes | Randomly selected | no | no | no | yes | no | no | yes | no |
| Measuring preference-based quality of life using the euroqol EQ-5D in patients with cerebral aneurysms. | Neurosurgery | 2009 | yes | Community dwelling | no | no | no | yes | yes | yes | no | no |

© 2020 Rebchuk AD et al. *JAMA Network Open*.

| | | | | | | | | | | | | |
|---|---|---|---|---|---|---|---|---|---|---|---|---|
| Recurrent stroke was associated with poor quality of life in patients with transient ischemic attack or minor stroke: Finding from the CHANCE trial. | CNS Neuroscience & Therapeutics | 2014 | yes | Randomly selected | no | no | no | yes | yes | yes | yes | no |
| The Stroke Impact Scale Version 2.0: Evaluation of reliability, validity, and sensitivity to change. | Stroke | 1999 | yes | Community dwelling | no | no | no | no | yes | no | yes | no |
| The combined impact of dependency on caregivers, disability, and coping strategy on quality of life after ischemic stroke. | Health & Quality of Life Outcomes | 2019 | no | Community dwelling | no | no | no | yes | no | yes | yes | yes |
| Quality of life and depression 3 months after intracerebral hemorrhage. | Brain and behavior | 2019 | yes | Community dwelling | no | no | yes | yes | yes | yes | yes | no |
| Utility-weighted modified Rankin Scale: Still too crude to be a truly patient-centric primary outcome measure?. | International Journal of Stroke | 2019 | yes | Randomly selected | no | yes | no | yes | yes | in original AVERT paper | yes | yes |
| PROMIS GH (Patient-reported outcomes measurement information system global health) scale in stroke a validation study. | Stroke | 2018 | yes | Community dwelling | no | yes | no | yes | yes | yes | yes | yes |

© 2020 Rebchuk AD et al. *JAMA Network Open*.
